# ElectroPen: An ultra-low–cost, electricity-free, portable electroporator

**DOI:** 10.1371/journal.pbio.3000589

**Published:** 2020-01-10

**Authors:** Gaurav Byagathvalli, Soham Sinha, Yan Zhang, Mark P. Styczynski, Janet Standeven, M. Saad Bhamla

**Affiliations:** 1 School of Chemical and Biomolecular Engineering, Georgia Institute of Technology, Atlanta, Georgia, United States of America; 2 Lambert High School, Suwanee, Georgia, United States of America

## Abstract

Electroporation is a basic yet powerful method for delivering small molecules (RNA, DNA, drugs) across cell membranes by application of an electrical field. It is used for many diverse applications, from genetically engineering cells to drug- and DNA-based vaccine delivery. Despite this broad utility, the high cost of electroporators can keep this approach out of reach for many budget-conscious laboratories. To address this need, we develop a simple, inexpensive, and handheld electroporator inspired by and derived from a common household piezoelectric stove lighter. The proposed "ElectroPen" device can cost as little as 23 cents (US dollars) to manufacture, is portable (weighs 13 g and requires no electricity), can be easily fabricated using 3D printing, and delivers repeatable exponentially decaying pulses of about 2,000 V in 5 ms. We provide a proof-of-concept demonstration by genetically transforming plasmids into *Escherichia coli* cells, showing transformation efficiency comparable to commercial devices, but at a fraction of the cost. We also demonstrate the potential for rapid dissemination of this approach, with multiple research groups across the globe validating the ease of construction and functionality of our device, supporting the potential for democratization of science through frugal tools. Thus, the simplicity, accessibility, and affordability of our device holds potential for making modern synthetic biology accessible in high school, community, and resource-poor laboratories.

## Abstract 2

This Community Page article describes an ultra-low–cost (23-cent) 3D-printed electroporator, inspired by a common barbecue lighter, designed to enable broader access to synthetic biology in high-school, community, and budget-conscious laboratories.

## Need for accessible electroporators in biology and bioengineering

Electroporators are used for a wide spectrum of purposes in molecular biology, biotechnology, and biomedical engineering [1,2]. Examples of these applications range from simple bacterial transformation [3] and eukaryotic transfection to more complex tasks, including genetic engineering with CRISPR [4], gene transfer into mammalian embryos [5], cancer treatments using electrochemotherapy [6], transdermal drug delivery [7], and gene-based vaccine delivery [8]. Despite these broad biological applications, commercial electroporators are complex and expensive pieces of bulky hardware that can cost thousands of dollars [9]. Their high cost places them out of reach for budget-restrained laboratories such as public high schools and research laboratories in resource-poor regions.

The need for accessible and affordable electroporators has previously prompted researchers to develop simple electronic circuits using relays and capacitors to amplify the voltage output required for electroporation [10–14]. However, many of these devices are still expensive and require extensive electronic components and hardware skills to construct, making them impractical for wide adoption. Beyond conventional power sources (batteries, etc.), piezoelectricity has been recognized as an alternative option to generate the large voltage pulses required and has been successfully applied to immunize mice [15,16]. Inspired by and building on the early success of piezoelectric-based electroporation, we set out to develop a simple, easy-to-fabricate, and low-cost electroporator (S1 Fig). Cognizant that commercial conducting cuvettes to hold liquid samples can often be a cost barrier, we also provide a simple method to build inexpensive millifluidic channels, using glass slides (or acrylic) with aluminum (or copper) tape, that can be used in place of cuvettes.

## Design and fabrication of ElectroPen

**Fig 1:**
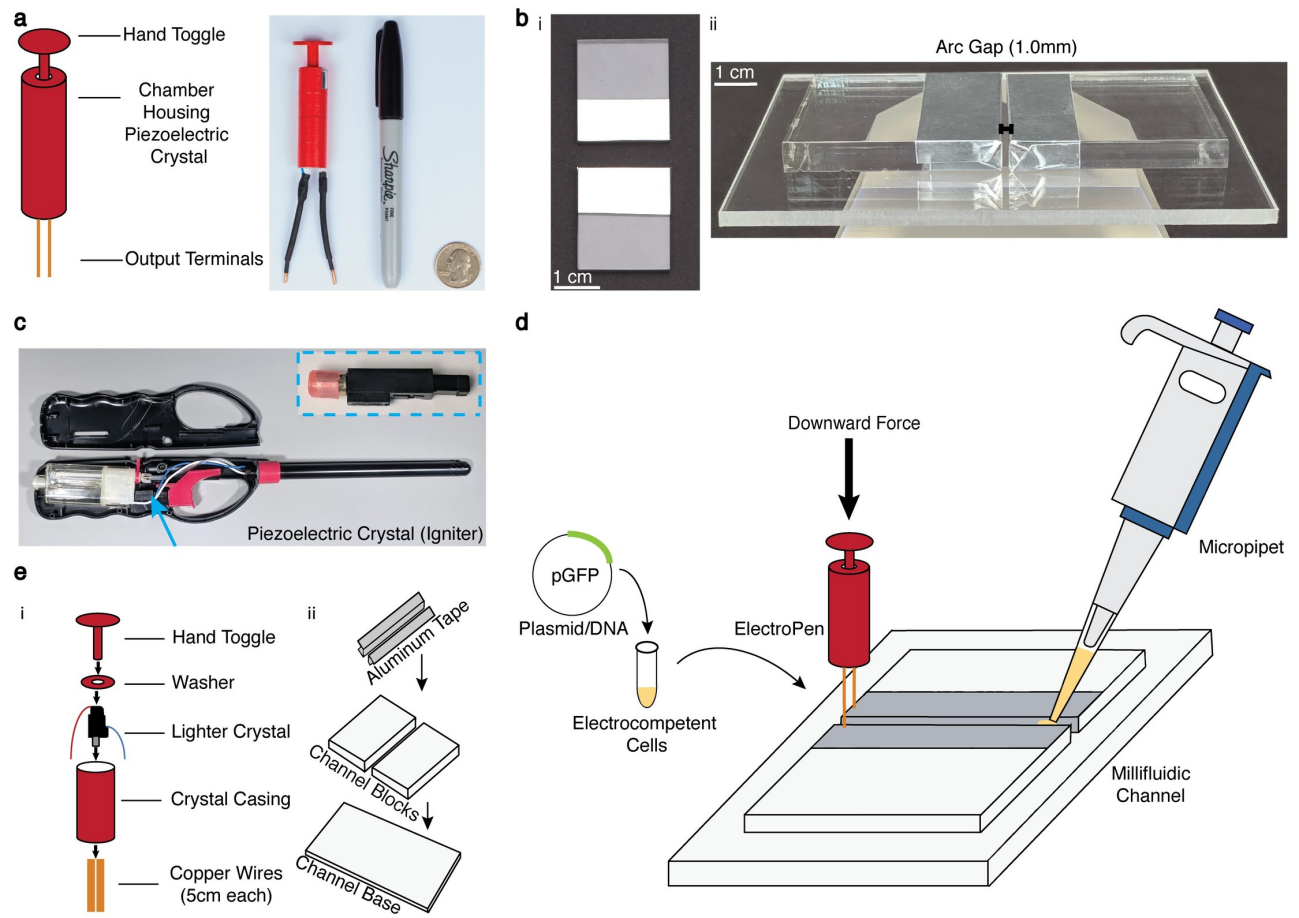
ElectroPen platform. (a) Design of the 3D-printed low-cost electroporation device along with a depiction of its size scale, demonstrating portability. The device is operated simply by pressing down the toggle to trigger the piezoelectric mechanism, resulting in electrical discharge. (b) Design of the alternative electroporation millifluidic channel. The millifluidic channel design consists of two blocks (shown here in acrylic) covered with aluminum tape to act as electrodes and placed on a base with a gap distance of 0.1 cm. The millifluidic channel can be built out of other materials (S5 Fig) as an alternative for industrial equivalents. (c) Depiction of the origin of the piezoelectric ignition mechanism found within the common stove lighter. The inset is the striker/piezoelectric mechanism of the lighter. The region with the red cap consists of a metal housing encasing the piezoelectric crystal. The middle black region consists of the spring–latch mechanism that strikes the crystal. The bottom black region (rightmost) consists of a wedge that is the origin for user-applied force and triggers the spring mechanism. The toggle on a lighter directly exerts a force on this mechanism to produce a spark. (d) Illustration of the general protocol for using the ElectroPen system. The cellular suspension is added to the gap in the millifluidic channel, after which the ElectroPen is connected and pressed to trigger a voltage potential. The cell suspension is then recovered in Luria Bertani broth and plated. See S1 Video for a detailed demonstration. (e) Illustration of the individual components of the 3D-printed ElectroPen platform and custom millifluidic channel. The CAD (.stl) file for the casing can be found on GitHub. CAD, computer-aided design; GFP, green fluorescent protein; pGFP, plasmid GFP.

The design of the ElectroPen includes a 3D-printed cylindrical chamber that houses a piezoelectric crystal harvested from a commercial lighter (Fig 1A and 1C). The chamber has wire passthroughs at the bottom and a hand toggle inserted at the top that, when pressed downwards, provides the equivalent force utilized in a conventional lighter (Fig 1D and 1E and S1 Video). The output voltage remains consistent independent of the user's force. The simplicity of the design provides for easy construction because an ElectroPen can be fabricated in 15 minutes (S2 Video and S2 Fig). With materials purchased in bulk, the ElectroPen can cost as little as 23 cents (US dollars) to make (Table 1).

**Table 1:** List of parts and costs for construction of the ElectroPen.

Part	Cost (in US Dollars)
Piezoelectric crystal	$0.02
Copper-plated wire	$0.10
Heat-shrinking wire insulator	$0.05
3D-printed casing	$0.05
Aluminum tape	$0.01
**ElectroPen**	**$0.23**

The net price is reflective of goods purchased in wholesale/large quantities from suppliers (excluding production costs). Links to the parts: https://www.alibaba.com/product-detail/Gas-Stove-Piezo-Igniter_846229760.html?spm=a2700.galleryofferlist.normalList.94.2a9c42f0IamSzp (piezoelectric crystal), https://www.alibaba.com/product-detail/8mm-Enamelled-bare-Copper-electrical-cable_60799942909.html?spm=a2700.7724838.2017115.53.7b8f95a3DwE73y (copper-plated wire), https://www.alibaba.com/product-detail/Custom-Insulated-Terminal-TC-200PC-Heat_60709731683.html?spm=a2700.galleryofferlist.normalList.73.69161808RjOrwX (heat-shrinking wire insulator), https://www.alibaba.com/product-detail/Competitive-advantages-3d-printing-black-pla_60520203080.html?spm=a2700.galleryofferlist.normalList.83.c5dd65ffTdLDAu (3D-printed casing), and https://www.alibaba.com/product-detail/High-Performance-Aluminum-Foil-Tape-for_1901874077.html?spm=a2700.galleryofferlist.normalList.234.29db24f8cC4A7P (aluminum tape).

Because of the high cost of standard electroporation cuvettes (for example, the list price of a sterile 0.1-cm electroporation cuvette is approximately $3–5 per cuvette [US dollars], with minimum package sizes often being 50 units), we develop a custom millifluidic channel that can be easily fabricated out of plastic (for example, acrylic) and for which custom gap-widths can be generated to accommodate different fluid volumes (Figs 1B and S3 and S4 and S5 and S3 Video). The general design of the millifluidic channel includes a base and two blocks with aluminum (or other conductive material such as copper) tape covering the sides of each block to function as electrodes and the space in between to hold the competent cell mixture to be transformed (Figs 1B and 1E and S3 and S1 Text). Although for our implementation, we use a glass slide or laser-cut acrylic design, we show that this technique can be easily extended to other materials such as wood (if appropriate sterility can be ensured), and reliable arc spacing of 0.1 cm can be achieved by using sheets of paper or a credit card to set the gap distance (S5 Fig and S3 Video).

## High voltage generation using piezoelectricity

**Fig 2:**
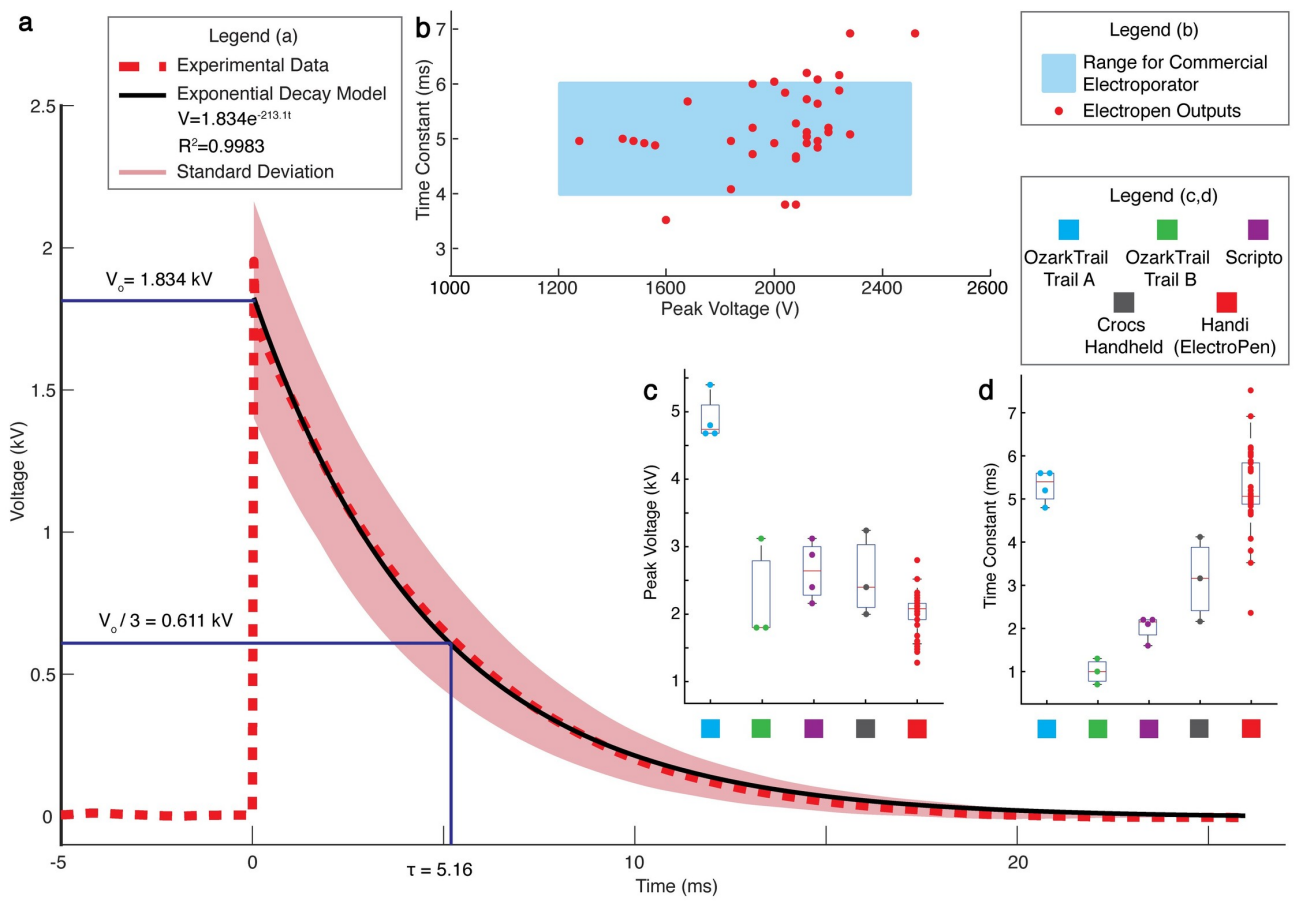
High-voltage output of the ElectroPen for electroporation. (a) Piezoelectric output from the ElectroPen is produced in the form of an exponentially decaying wave (the optimal waveform for electroporation of *E*. *coli* cells [17],) achieving an average peak voltage output of *V*_max_ = 2 ± 0.3 kV and time constant *τ* = 5.1 ± 0.9 ms. The average curve is calculated from *n* = 38 firings by 3 users (smoothed using a Lowess regression model with a span of 12%), with the shaded area indicating the standard deviation. The raw unsmoothed data for all the outputs are provided in S12 Fig. V_0_ indicates the average initial value of the waveforms, and the time constant is defined as the time taken for the waveform to decay from its peak voltage to one-third of its peak voltage. This data set was obtained using the Handi lighter (Fayco Industries, New Brunswick, NJ, USA; same lighter used to conduct trials for Fig 4A and 4B). (b) The maximum voltage and time constant outputs produced by the ElectroPen are within the range of commercial electroporators (optimized for *E*. *coli*) [18]. From [18], the recommended field strength range is 12–25 kV/cm, and the corresponding peak voltages are 1.2–2.5 kV (for a 0.1-cm arc gap). The optimal time constant range has also been indicated as between 4–6 ms. Data represent *n* = 38. (c) Average peak voltage produced by different brands of lighters, demonstrating the range of values possible by purchasing lighters from different companies. Data represent *n* = 4, 3, 4, 3, and 38, respectively. (d) Average time constants produced by different brands of lighters. Data represent *n* = 4, 3, 4, 3, and 38, respectively. For c and d, *n* ≥ 3 measurements are used for each brand, and the lines represent the median of the data set; the edges of the box represent the quartiles, with the bottom representing 25th and top representing 75th percentiles; the whiskers extend to the most extreme data points not considered outliers; and for normally distributed data, whiskers correspond to approximately ±2.7*σ*, where *σ* is the standard deviation. The data for a, b, c, and d can be found on GitHub under the S1 Data file, under the sheets titled Fig 2A, 2B and 2C and 2D, respectively.

We next measure the electrical response of the ElectroPen using a high-voltage probe connected to an oscilloscope. The average curves (of *n* = 38 firings from 3 users) follow an exponential function described by V(t) = *V*_max_*e*^(−*αt*)^, where function V is the voltage in kV, t is the time in seconds, *V*_max_ is the initial maximum voltage in kV, and *α* = 1/*τ* is the exponential decay time constant to reach one-third of the initial value measured in s^−1^, as shown in Fig 2A. The exponential decay is a function of the piezoelectric effect occurring through the polarization of the lead zirconate titanate (PZT) crystal. We note that the electrical output is remarkably reproducible over many trials conducted by multiple users; this is due to the spring-based design of the ElectroPen, which is discussed in greater detail in the next section. Although successful electroporation can be achieved with a wide range of voltages, optimal transformation efficiency occurs over a narrow range [17]. Thus, a spring-based mechanism enables consistent voltage output that is tunable by the crystal properties but independent of the force applied by a user on the ElectroPen.

We evaluated a number of different lighter crystals (Fig 2B, 2C and 2D) and selected one that would produce a maximum voltage of *V*_max_ = 2 ± 0.3 kV with an average time constant *τ* = 5.1 ± 0.9 ms (Fig 2A and 2B). These values correspond to a field strength of 20 kV/cm, which is optimal for *E*. *coli* transformation [17,18]. Although the current voltages and time constants are within the range of values for successful electroporation of *E*. *coli* as shown in Fig 2B, we demonstrate that the underlying principle of this device can be used to generate *V*_max_ = 30,000 V (S6 Fig) and tuned (by choosing between crystals with different outputs) for a range of time constants and voltage outputs for different biological and biomedical applications (S2 Table).

## Reproducible voltages generated through a simple spring-latch mechanism

**Fig 3:**
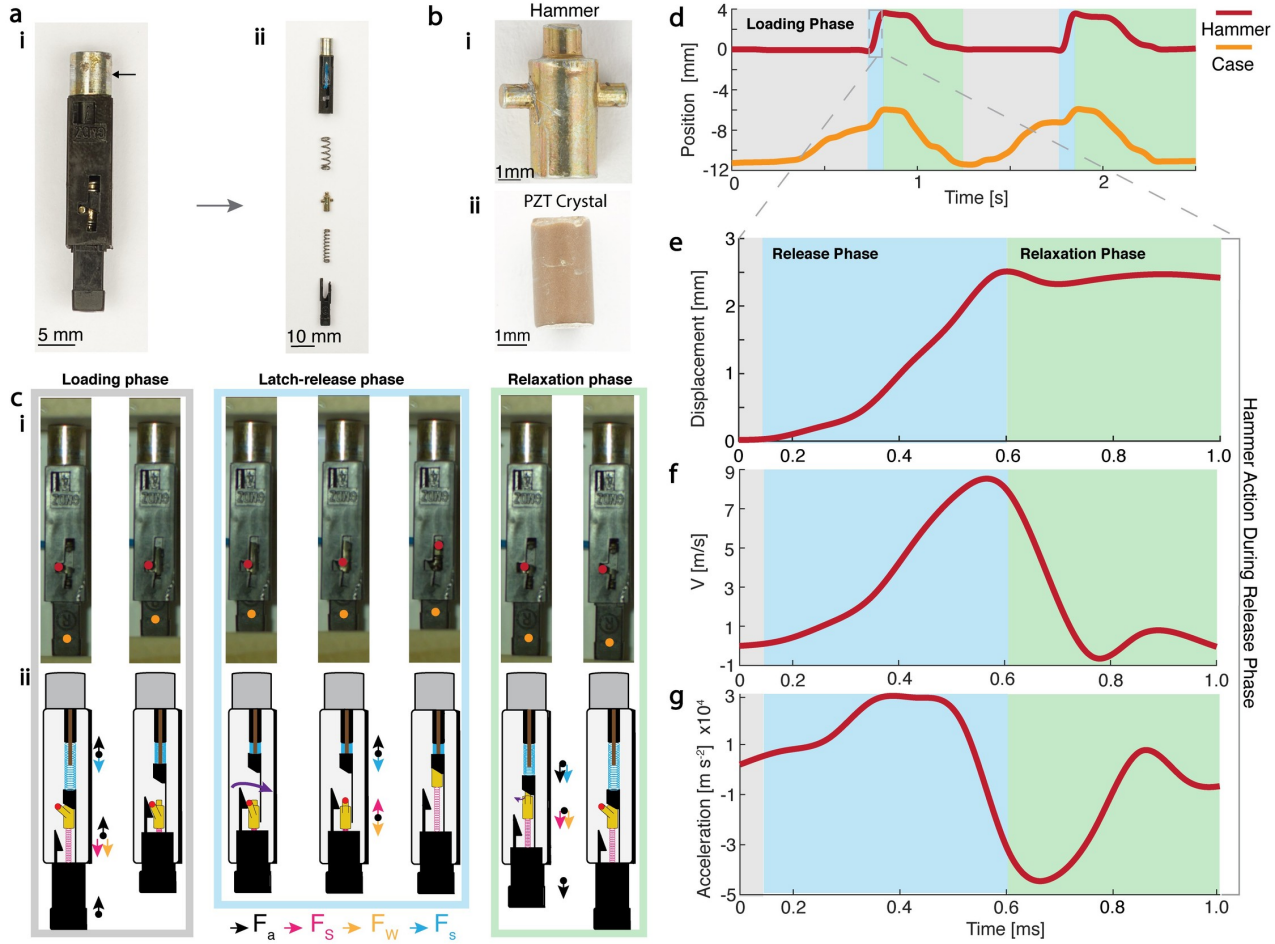
Spring-latch mechanisms for repeatable generation of high-voltage pulses. (a) (i) Image of the striking mechanism (hammer action) found within the piezo igniter in a lighter (arrow indicates location of crystal). (ii) The parts include, from top to bottom, metal conductor (gold-colored region) housing the piezoelectric crystal, springs, hammer, release spring, and geometrical latch. The presence of two springs is to decouple the loading and release phase for consistent voltage output. (b) Images of the hammer and PZT crystal. The circular surface area of the hammer comes into direct contact with a pin that strikes the piezoelectric crystal, generating a voltage through the piezoelectric effect. (c) (i) Snapshots from high-speed video illustrating the position of the hammer during the loading, latch-release, and relaxation phases (S4 Video). (ii) Free-body diagram indicating movement of each part through each phase of the hammer action, including activation and deactivation of spring forces. (d) Plot of displacement of the hammer and the lower case as a function of time obtained using high-speed image video. (e–g) Zooming into the dynamics of the hammer during the latch-release phase reveals that the hammer achieves a peak velocity of 8 ms^−1^ in 0.5 ms, which corresponds to an acceleration of 30,000 ms^−2^. The explosive acceleration results in a 10-N force (mass of hammer is 0.3 g) exerted over a tiny area of the PZT crystal. The data for d–g can be found on GitHub under the S2 Data file, under the sheets titled Fig 3D, 3E and 3F and 3G, respectively. PZT, lead zirconate titanate.

One of the key reasons why the ElectroPen can be so useful for bacterial transformation applications is because it can generate repeatable, consistently high voltages independent of user force. The ElectroPen exploits a simple and inexpensive mechanical spring-latch mechanism to release a small hammer-pin structure onto the piezoelectric crystal to generate high voltages, rather than the complex electrical circuits with costly microprocessor-controlled relays that commercial electroporators use to generate similar voltages. This spring-latch mechanism was consistent across almost 20 lighters from different commercially available brands that we disassembled and inspected. To illustrate the underlying mechanics, we recorded the rapid motion of the piezoelectric crystal using a high-speed camera at 1,057 frames per second (S4 Video). The mechanism consists of two springs, a hammer (metal piece striking the crystal), and the PZT crystal itself connected to a metal conductor (Fig 3A and 3B). The hammer action functions in 3 phases: a loading phase, latch-release phase, and a relaxation phase (Fig 3C–3E).

During the loading phase, the hammer is held in a locked position by a mechanical latch as the lower spring and upper spring are being compressed. This phase generates spring potential energy by compressing both springs through the user-exerted force on the 3D-printed hand toggle. As the compression continues, the wedge-shaped piece of the casing forces the pin of the hammer to rotate outwards of the latch. Once the pin of the hammer has reached a critical point in its rotation, i.e., it is no longer held in place by the latch, the lower spring instantaneously decompresses. This act converts the stored spring energy into the kinetic energy of the hammer, resulting in a high impact force on the cylindrical face of the PZT crystal to generate voltage. Since the degree of lower spring compression is dependent only on the spring–latch design, the quantity of force striking the crystal by the hammer is independent of the user-applied force on the toggle. As a result, the output voltages are remarkably consistent. In the relaxation phase, the user releases the user-applied force to reset the hammer to its initial position.

Analysis of high-speed videos of the hammer releasing indicates that the hammer is able to reach a maximum velocity of 8 m/s at a peak acceleration of almost 30,000 ms^−2^, a force equivalent to 3,000 g-force (Fig 3E–3G). Through the explosive nature of the hammer action's acceleration, a powerful resultant impulsive force of 10 N strikes the PZT crystal, resulting in a high-voltage pulse. Using this value of force and parameters of the PZT crystal, we can theoretically predict the voltage output as *V*_*th*_ = 2.7 kV (S2 Text), which is on the same order of magnitude as the experimentally measured voltages shown in Fig 2.

## Transformation of *E*. *coli* using ElectroPen

**Fig 4:**
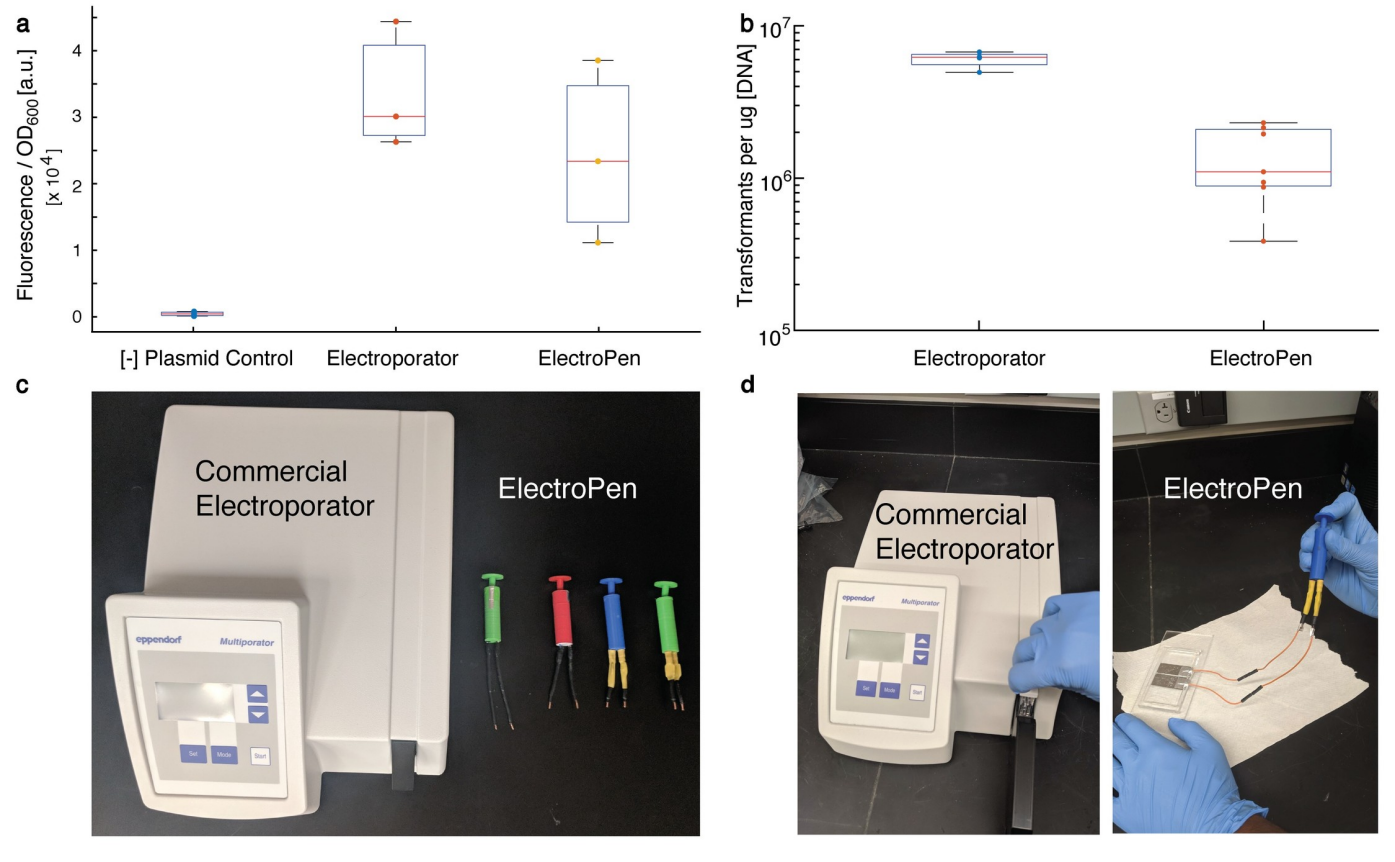
*E*. *coli* transformation using ElectroPen. (a) Fluorescence output due to expression of GFP from a transformed plasmid is similar between a commercial electroporator and the ElectroPen, confirming successful transformation and GFP expression. Here, [−] plasmid control refers to electroporation with no plasmid DNA as a reference measurement for fluorescence. Data represent *N* = 3 trials (see S1 Text). (b) Transformation efficiencies for the commercial electroporator (Bio-Rad MicroPulser) and ElectroPen are within an order of magnitude (see inset). Data from *N* = 4 for electroporator and *N* = 7 for ElectroPen. A negative control of the same plasmid DNA and electrocompetent cells without electroporation yielded zero colonies on selective media for all attempts. For a and b, the lines represents the median of the data set; the edges of the box represent the quartiles, with the bottom representing 25th and top representing 75th percentiles; the whiskers extend to the most extreme data points not considered outliers; and for normally distributed data, whiskers correspond to approximately ±2.7*σ*, where *σ* is the standard deviation. (c) Image of a commercial electroporator alongside ElectroPens. (d) Image of cuvette being inserted into electroporator for usage alongside usage of the ElectroPen (with hardwired connections). The data for a and b can be found on GitHub under the S3 Data file, under the sheets titled Fig 4A and 4B, respectively. a.u., arbitrary unit; GFP, green fluorescent protein; OD, optical density.

To demonstrate the utility of the ElectroPen in enabling bacterial electrotransformations, we prepare *E*. *coli* (BL21 strain) electrocompetent cells and transform them with a recombinant plasmid with superfolder green fluorescent protein (sfGFP) expression under the control of a constitutive promoter (J23119 from the Parts Registry; see S1 Text and S7 Fig). Electroporation is performed using both the ElectroPen with a custom 0.1-cm millifluidic channel and a commercial electroporator with a 0.1 cm electroporation cuvette (Bio-Rad MicroPulser; Hercules, CA, USA). Measured green fluorescence protein (GFP) fluorescence levels confirm that the plasmid encoding GFP is successfully electroporated using the ElectroPen (Fig 4A) in comparison to the negative control (electroporation with no plasmid DNA). We note that this fluorescence experiment is to confirm that the electroporation process did not damage the DNA being transformed or cause undue plasmid instability in the electroporated cells. To confirm that the transformation was indeed due to electroporation and not by natural competence of the cells, a second negative control of the same plasmid DNA and electrocompetent cells without electroporation was conducted, which yielded zero colonies on selective media for all attempts. The fluorescence intensity values for the ElectroPen trials are within the range of outputs produced by the standard electroporator. Moreover, the transformation efficiencies between the ElectroPen and the standard electroporator are within an order of magnitude (Fig 4B).

Finally, we highlight the potential impact of the ElectroPen by demonstrating how it can be rapidly disseminated and used for synthetic biology experiments via collaboration with two teams from the International Genetically Engineered Machine (iGEM) competition. We shared the device design files, sample protocols, and digital instructions with the University of Georgia (UGA) and Taipei American School (TAS) (Taiwan) iGEM teams (S10 Fig). These teams of high school students and undergraduates tested the ElectroPen by transforming plasmids encoding GFP expression into two different *E*. *coli* strains, DH5*α* (UGA and TAS Taipei) and Nissle 1917 (TAS Taipei). The teams obtained successful fluorescence expression and comparable transformation efficiency data, validating the reproducibility and rapid dissemination of the ElectroPen (S8 and S9 and S10 Figs).

## Advantages and limitations of the ElectroPen

Electroporators are versatile tools for genetic engineering and basic biology. A new push towards frugal science has inspired the development of many low-cost and open source scientific devices [19–26] that allow expansion of these disciplines into high schools, underfunded laboratories, and even field research. These low-cost devices serve as alternatives for expensive lab equipment while simultaneously removing numerous barriers even beyond cost, including access to electricity and portability. Here, we have theoretically modeled and experimentally validated the functionality and effectiveness of the ElectroPen, the cheapest electroporator in the world (Fig 4C and 4D and S1 Table). A key component of the electroporator is a piezoelectric crystal that can be obtained from common stove lighters that can be easily purchased in most parts of the world (S13 Fig). While the ElectroPen serves as a suitable alternative for commercial electroporators, a device at this cost comes with a few disadvantages that we outline below.

The first involves variability in the piezoelectric crystals found in lighters, both from batch to batch and different brands. Peak voltages (*V*_0_) and time constants (*τ*) are dependent on the type of crystal material, the dopants utilized, and the company from which it was purchased, which we demonstrate in Fig 2C and 2D, and therefore, not every lighter crystal is suitable for electroporation of *E*. *coli*. In fact, we are currently unable to obtain more crystals with the same parameters as the crystals used for all the transformation experiments described here (from Handi lighters, manufactured by Fayco Industries) because of this variability in characteristic parameters. Other lighters from many different brands that we bought from other stores (Walmart, Amazon, Target) did not provide the required combination of voltages and time constants. For example, the Ozark Trail A shown in Fig 2B produces a time constant of 5 ms but a peak voltage of 5 kV, making it unsuitable for *E*. *coli* transformation. For other users, we recommend that they first test the crystal they obtain from a lighter using the protocol described in S1 Text to quantify the pulse produced by the crystal to ascertain whether it is suitable for their specific biological system. The second is that the piezoelectric crystals have a limited life span. The life span of the crystal is based on the number of times the crystal is hit; however, this can vary between sellers because of the dopants used in the piezoelectric crystal. Typically, the crystal life is mentioned on the front or back cover of purchased lighters. Third, no optical indicator exists on our setup to indicate that a pulse was generated and applied to the sample during the trial. Successful electroporation was determined by evaluating growth of bacteria on an antibiotic resistance plate, which takes time. Thus, it can become challenging to quickly debug unsuccessful pulse transmission caused by either user error or instrument malfunction until after cells are cultured. One possible solution is to connect a light-emitting diode (LED) light in parallel to the conductive path for electroporation, serving as a visual indicator (S14 Fig) for passage of the pulse through the cell suspension. Fourth, an improper contact between the ElectroPen wires and the millifluidic electrodes can prevent establishment of the electric potential necessary for successful transformation. To minimize these contact mismatch errors between the conductive surface and improve user friendliness, hardwired connections can be employed, as shown in Fig 4D.

Despite these minor shortcomings, which we hope to address in future iterations, our work directly opens up avenues for hands-on biology experiments in high schools and budget-conscious laboratories. Ultimately, the ElectroPen is another example in frugal science that serves to bypass economic and infrastructure limitations in the advancement of scientific research by the next generation of young scientists across the globe.

## Supporting information

S1 Fig Advantages and disadvantages of ElectroPen. Workflow schematic for usage and applications of the ElectroPen in comparison to commercial electroporators, as well as their advantages and drawbacks.
(TIF)

S2 Fig Illustration of the assembly of the ElectroPen. This depiction indicates the overall construction process with a tutorial found in S2 Video.
(TIF)

S3 Fig Illustration of the assembly of the ElectroPen cuvette. This depiction indicates the overall construction process with a tutorial found in S2 Video.
(TIF)

S4 Fig Size comparison between the ElectroPen and commercial electroporator. (a) Difference in size and weight between the ElectroPen and Bio-Rad MicroPulser. (b) Difference in size between commercial electroporation cuvette and ElectroPen cuvette built using a glass slide.
(TIF)

S5 Fig Depiction of the different variants of the cuvette built using the described basic principles. (a) Pictured left to right are the electroporation cuvette, glass slide cuvettes, and acrylic cuvette. (b) Image of the cuvette used to run the majority of trials in comparison with the commercial cuvette. (c) Cuvette built using wooden blocks. (d) Parts of the acrylic block, with the left piece being the extra surrounding material following laser cutting.
(TIF)

S6 Fig ElectroPen waveforms. Waveforms produced by ElectroPen using different piezoelectric crystals demonstrating voltage in excess of 25,000 Volts.
(TIF)

S7 Fig Plasmid map. Diagram depicting the plasmid map of the pADS001 plasmid utilized in the trials conducted.
(TIF)

S8 Fig Transformation efficiency data from the trial conducted at the UGA using DH5a *E*. *coli*. The data for S8 Fig can be found on GitHub under the S4 Data file, under the sheet titled S8 Fig. UGA, University of Georgia.
(TIF)

S9 Fig Images of the plates with fluorescence from the independent collaboration trials conducted by TAS Taipei iGEM 2018. iGEM, International Genetically Engineered Machine; TAS, Taipei American School.
(TIF)

S10 Fig Images of students from different iGEM teams across the world testing the ElectroPen. iGEM, International Genetically Engineered Machine.
(TIF)

S11 Fig Setup utilized to quantify voltage output. (a) The oscilloscope was connected to a high-voltage probe along with the ElectroPen. (b) Oscilloscope connections to the cuvette while running the trials to ensure voltage passed through cell suspension. (c) Sample waveform obtained from oscilloscope during trial (voltage clipping is present as ElectroPen voltage output exceeds capacity of the oscilloscope).
(TIF)

S12 Fig Raw data for voltage pulse values presented in Fig 2A. The solid black line represents the average value for the decay pulse (without curve fitting), and the scatter points represent individual values. The data for S12 Fig can be found on GitHub under the S1 Data file, under the sheet titled Fig 2A.
(TIF)

S13 Fig Lighters are common, as illustrated by images obtained from various sites selling these in different countries (links in inset). Indicative of how the overall mechanism and construction of lighters remains consistent across the world.
(TIF)

S14 Fig LED indicator design. (a) Potential setup for LED light indicator to provide identification for successful electroporation. (b) Potential copper electrodes for millifluidic channels instead of aluminum tape electrodes. (c) Potential hardwired version of ElectroPen. LED, light-emitting diode.
(TIF)

S1 Video Protocol for usage of the ElectroPen. Video outlining the overall method for using the ElectroPen. The cuvette for electroporation was kept in a −20°C freezer for 24 hours prior to electroporation and held on ice prior to conducting the trial.
(MOV)

S2 Video Construction process for ElectroPen device. Video outlining the overall method for the construction of the ElectroPen. Video speed has been increased, but the average time taken to build an ElectroPen is approximately 15 minutes.
(MP4)

S3 Video Construction process for the millifluidic channel channel. Video outlining the overall method for the construction of the ElectroPen cuvette. Video speed has been increased, but the average time taken to build an ElectroPen cuvette is approximately 5 minutes.
(MOV)

S4 Video High-speed dynamics of the hammer action. High-speed video (1,057 fps) of the ElectroPen's hammer action proceeding through the described 3 stages: loading phase, latch-release phase, and relaxation phase. During the loading phase, as the user applies an input force, the casing moves upwards, compressing the lower and upper spring while the hammer remains in the locked position. The applied force results in a wedge pushing the hammer arm out of the latch (locking position). After the hammer arm has completely moved out of the latch, the latch-release phase begins, and the spring extends, projecting the hammer upwards to strike a pin connected to the piezoelectric crystal. The hammer then returns to the original position as the user pulls back on the hammer action, restoring the original position.
(MOV)

S1 Text Materials and methods. (PDF)

S2 Text Discussion of theoretical predication of voltage from piezoelectric crystals. (PDF)

S1 Table Comparison between standard commercial electroporators and the ElectroPen. The listed electroporators reflect common equipment utilized in labs. The ElectroPen reflects a fraction of the cost of its industrial equivalent while not requiring access to electricity and weighing magnitudes less. *0.1/0.2-cm gap industrial electroporation cuvettes. †The cost includes only the device.
(PDF)

S2 Table List of different time constants and peak voltages optimized for the electroporation of different organisms. The protocols for electroporation for these organisms can be found at the respective links attached.
(PDF)

## Abbreviations

a.u.: arbitrary unit
CAD: computer-aided design
GFP: green fluorescent protein
iGEM: International Genetically Engineered Machine
LED: light-emitting diode
OD: optical density
pGFP: plasmid GFP
PZT: lead zirconate titanate
sfGFP: superfolder GFP
TAS: Taipei American School
UGA: University of Georgia
