# Supplementary figures and images for: ElectroPen: An ultra-low–cost, electricity-free, portable electroporator
Source: PLoS Biol. 2020 Jan 10;18(1):e3000589. doi: 10.1371/journal.pbio.3000589 (PMC6953602; doi:10.1371/journal.pbio.3000589)

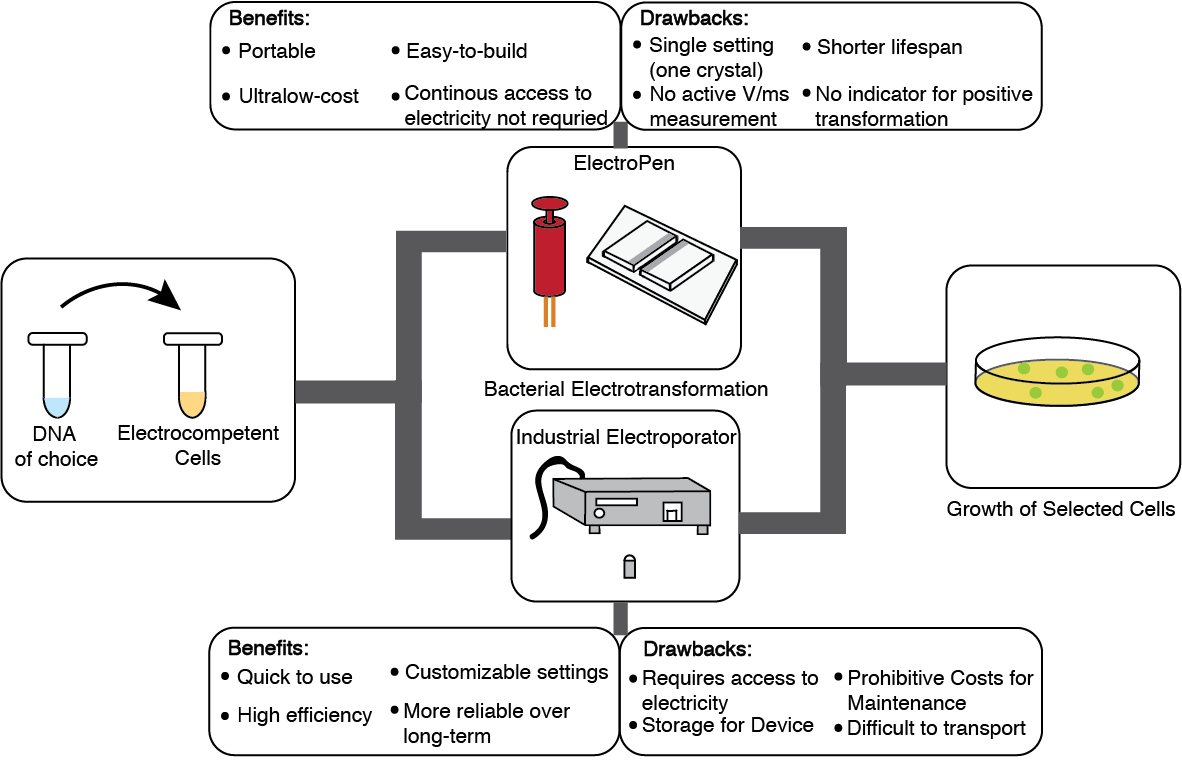

Supplement: S1 Fig — Workflow schematic for usage and applications of the ElectroPen in comparison to commercial electroporators, as well as their advantages and drawbacks. (TIF) [file pbio.3000589.s001.tif]

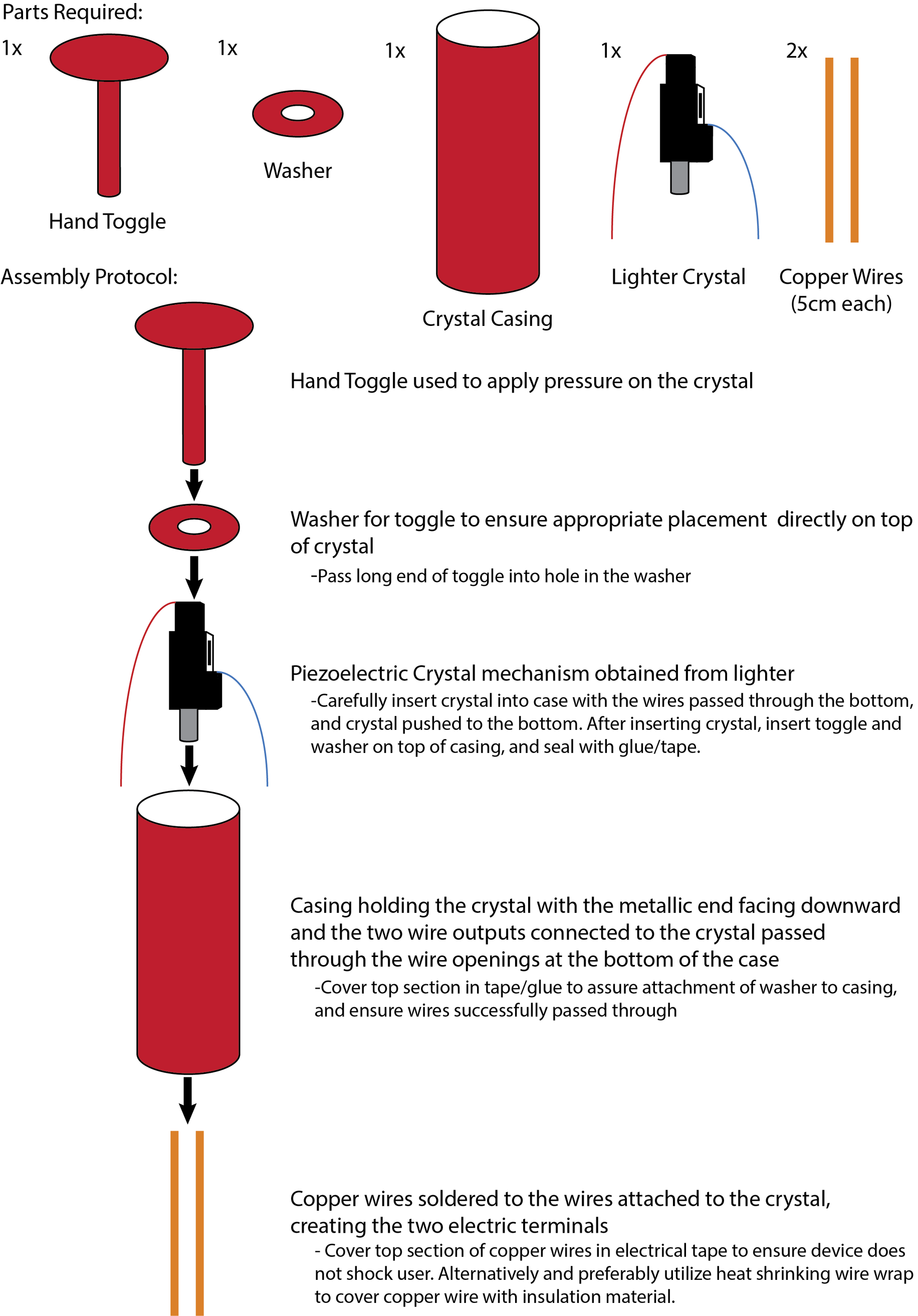

Supplement: S2 Fig — This depiction indicates the overall construction process with a tutorial found in S2 Video. (TIF) [file pbio.3000589.s002.tif]

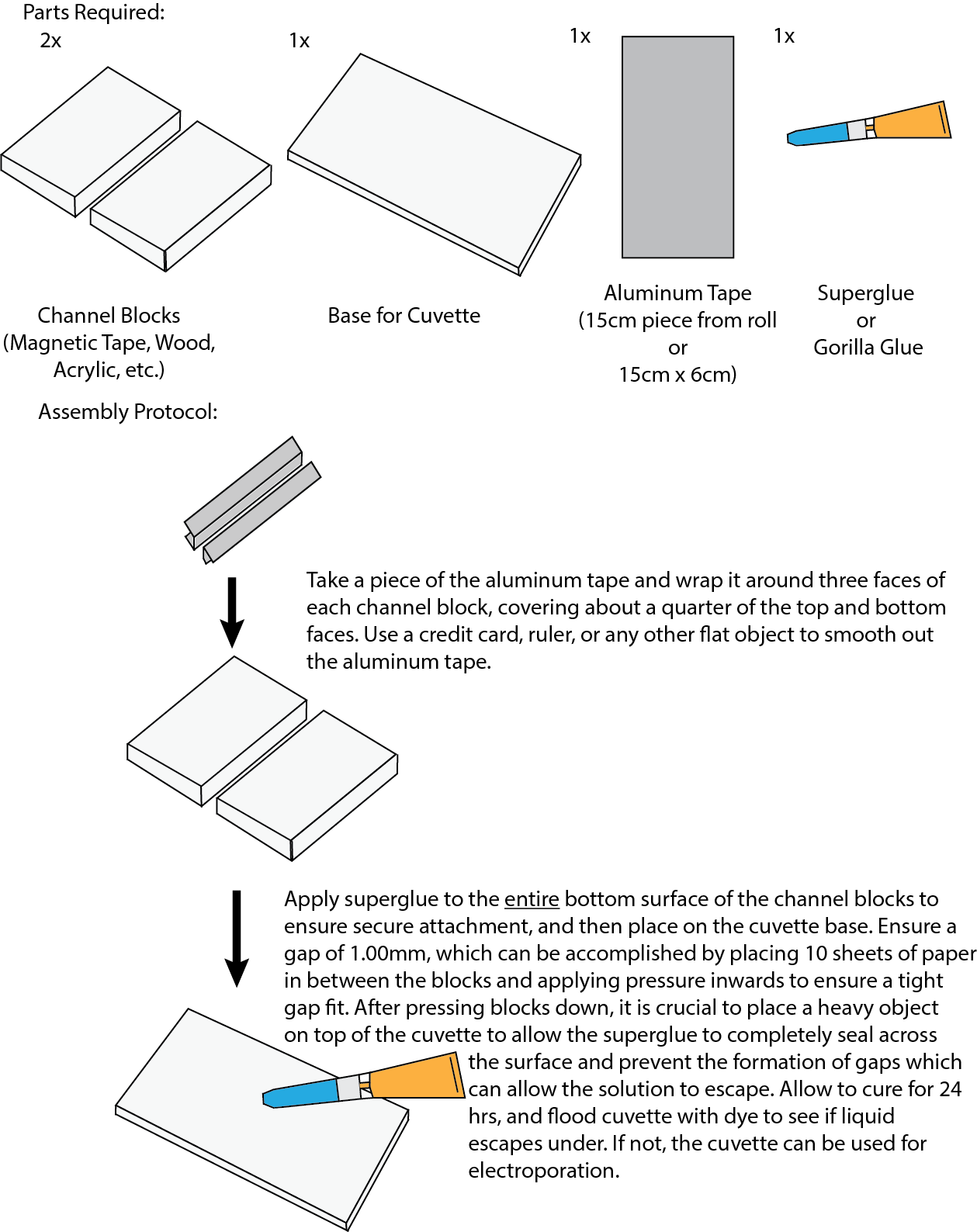

Supplement: S3 Fig — This depiction indicates the overall construction process with a tutorial found in S2 Video. (TIF) [file pbio.3000589.s003.tif]

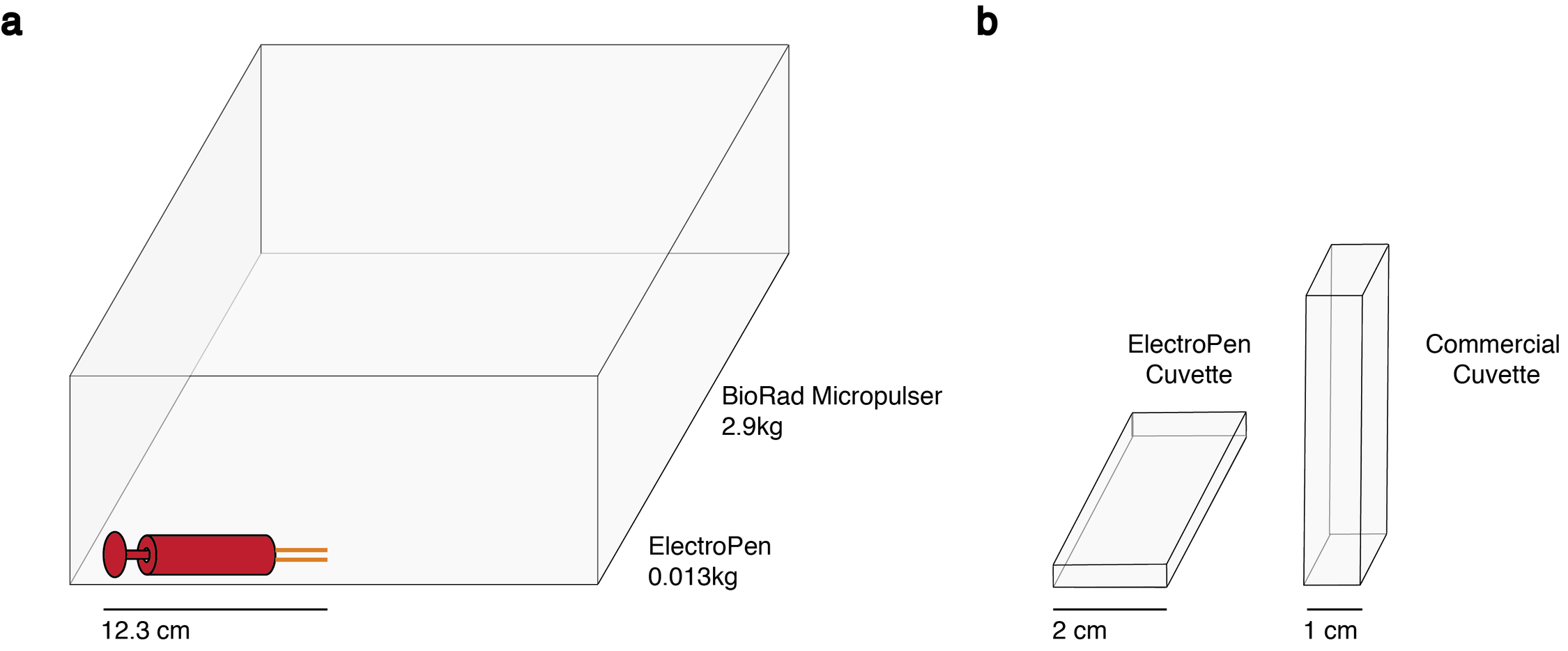

Supplement: S4 Fig — (a) Difference in size and weight between the ElectroPen and Bio-Rad MicroPulser. (b) Difference in size between commercial electroporation cuvette and ElectroPen cuvette built using a glass slide. (TIF) [file pbio.3000589.s004.tif]

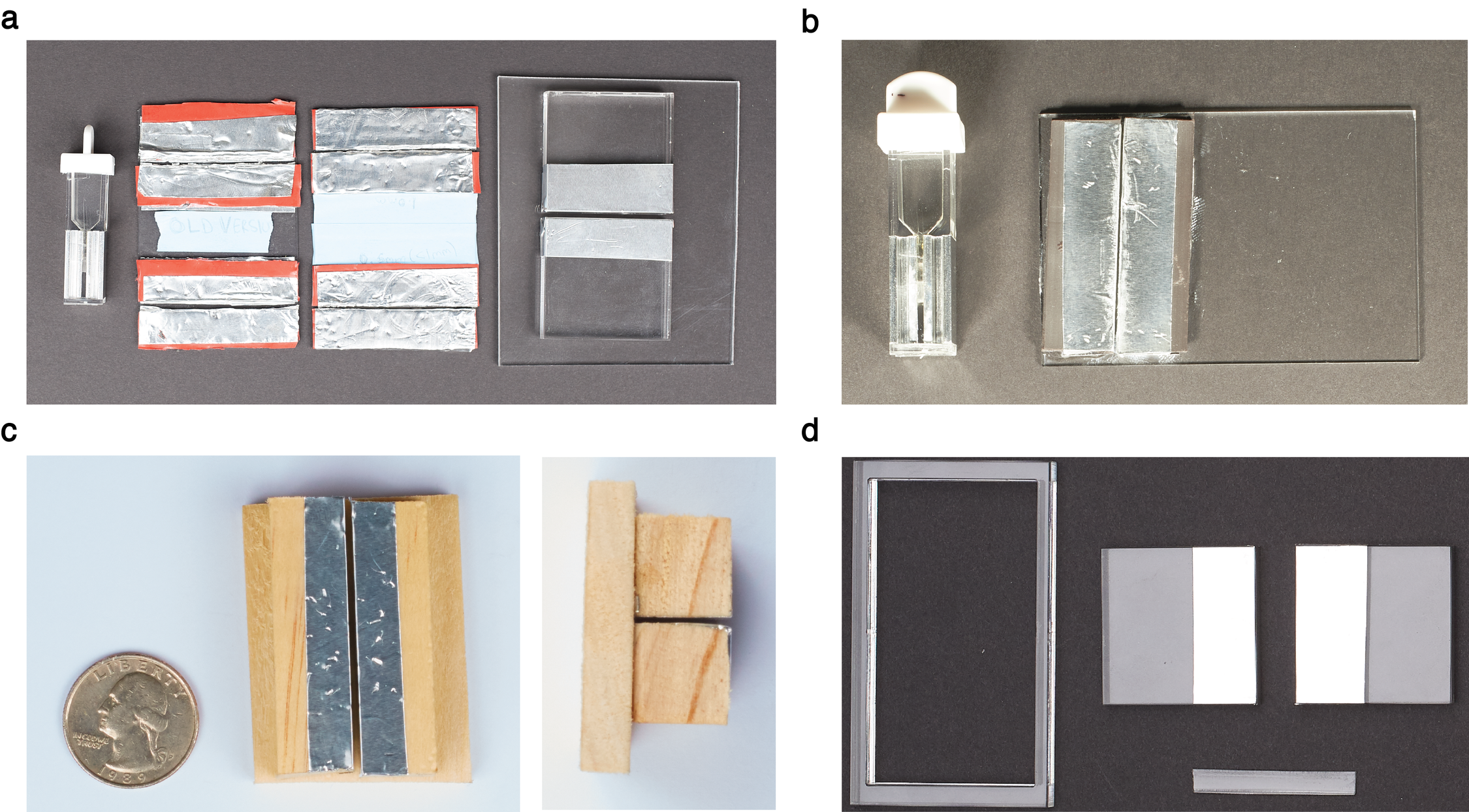

Supplement: S5 Fig — (a) Pictured left to right are the electroporation cuvette, glass slide cuvettes, and acrylic cuvette. (b) Image of the cuvette used to run the majority of trials in comparison with the commercial cuvette. (c) Cuvette built using wooden blocks. (d) Parts of the acrylic block, with the left piece being the extra surrounding material following laser cutting. (TIF) [file pbio.3000589.s005.tif]

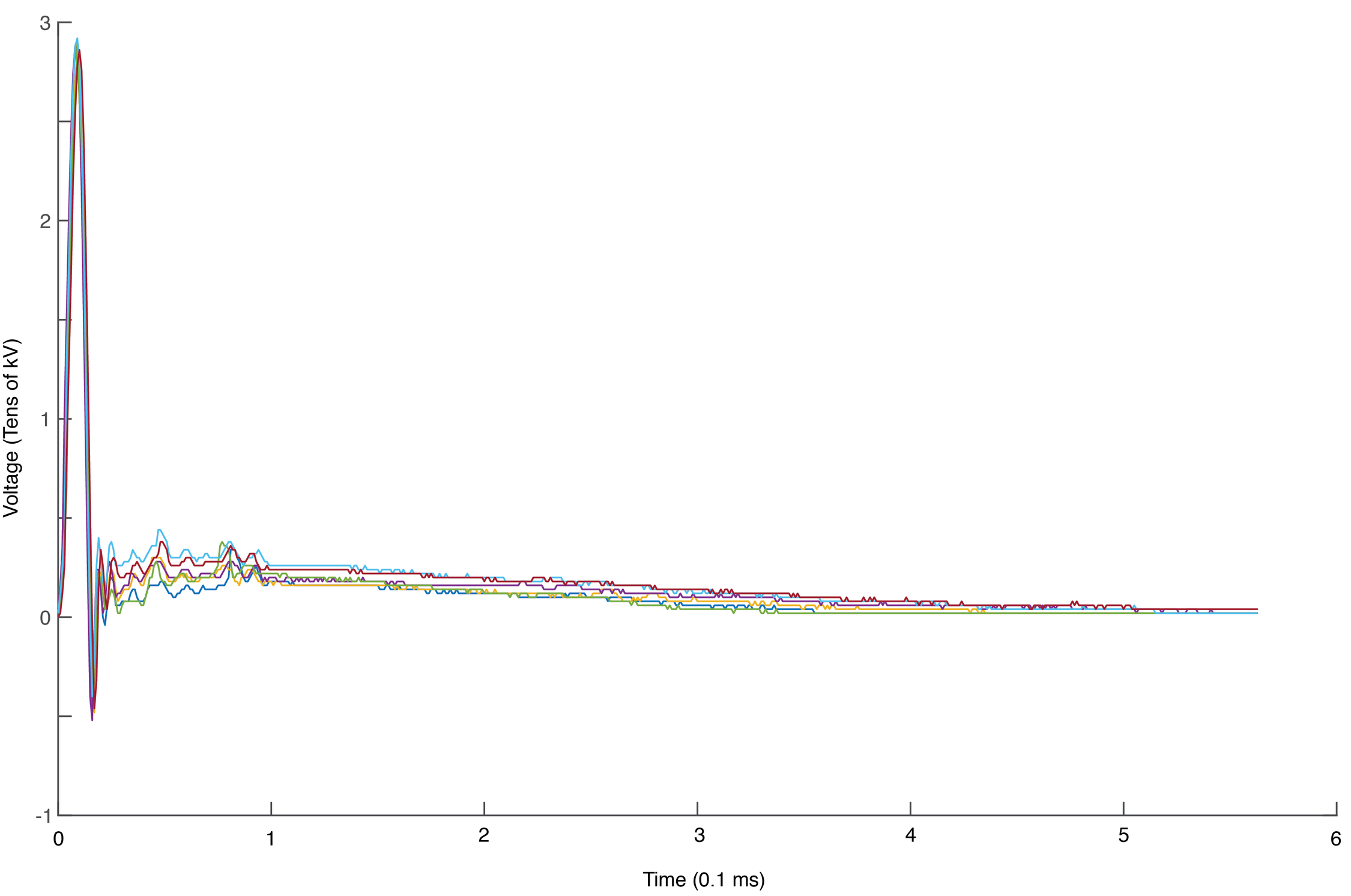

Supplement: S6 Fig — Waveforms produced by ElectroPen using different piezoelectric crystals demonstrating voltage in excess of 25,000 Volts. (TIF) [file pbio.3000589.s006.tif]

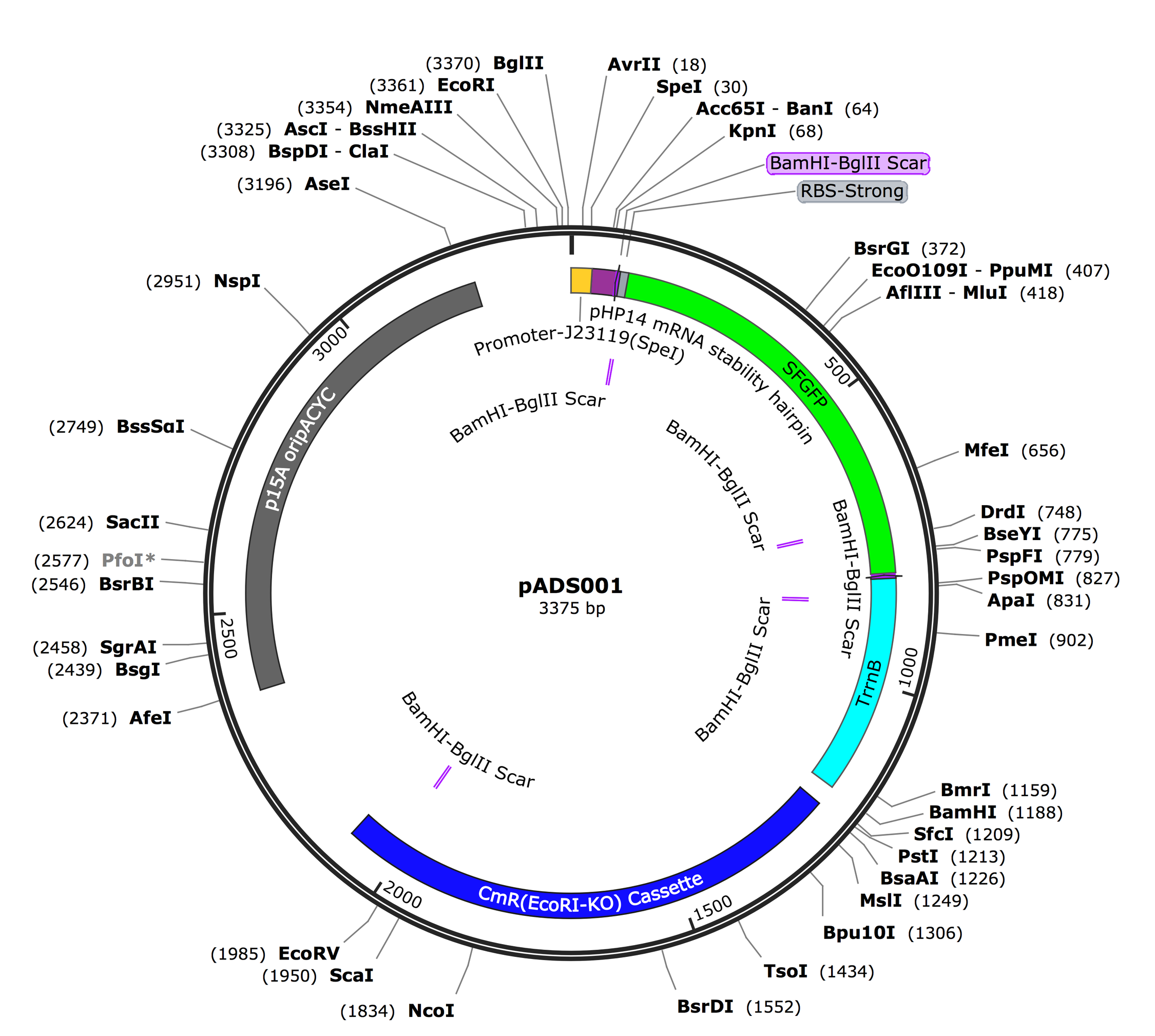

Supplement: S7 Fig — Diagram depicting the plasmid map of the pADS001 plasmid utilized in the trials conducted. (TIF) [file pbio.3000589.s007.tif]

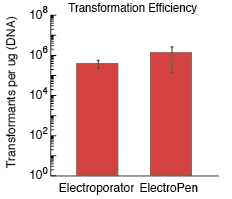

Supplement: S8 Fig — The data for S8 Fig can be found on GitHub under the S4 Data file, under the sheet titled S8 Fig. UGA, University of Georgia. (TIF) [file pbio.3000589.s008.tif]

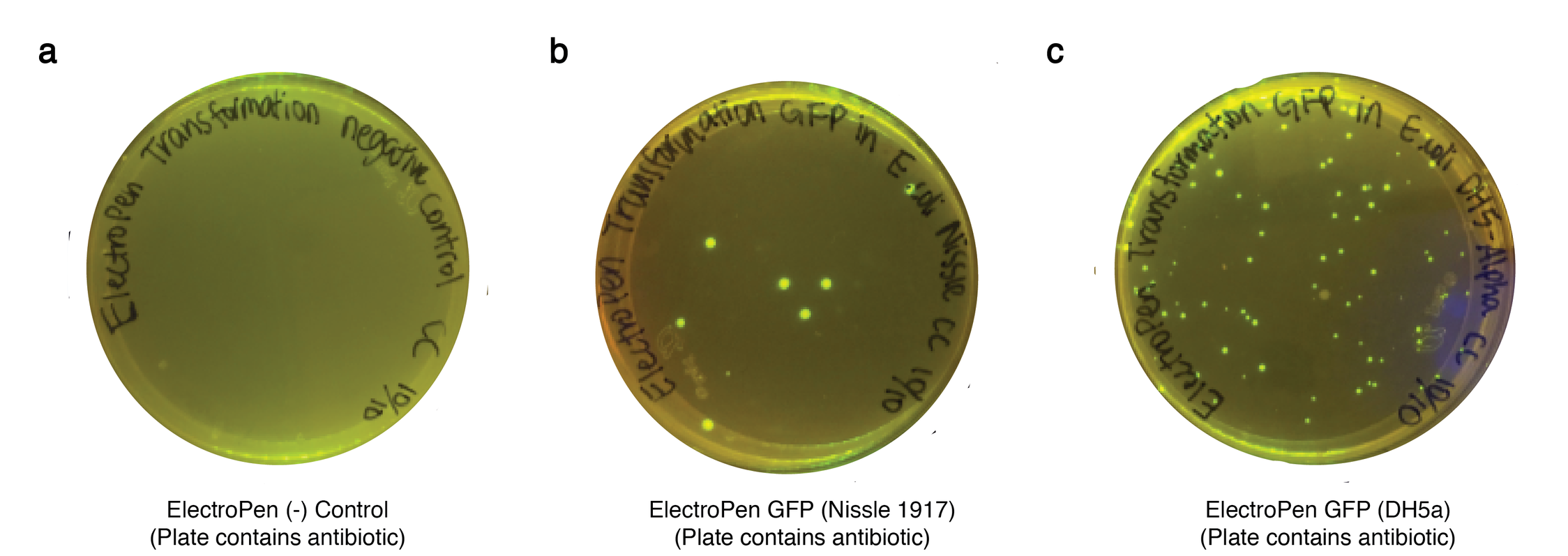

Supplement: S9 Fig — iGEM, International Genetically Engineered Machine; TAS, Taipei American School. (TIF) [file pbio.3000589.s009.tif]

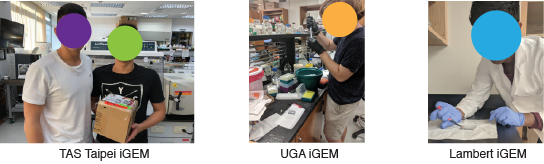

Supplement: S10 Fig — iGEM, International Genetically Engineered Machine. (TIF) [file pbio.3000589.s010.tif]

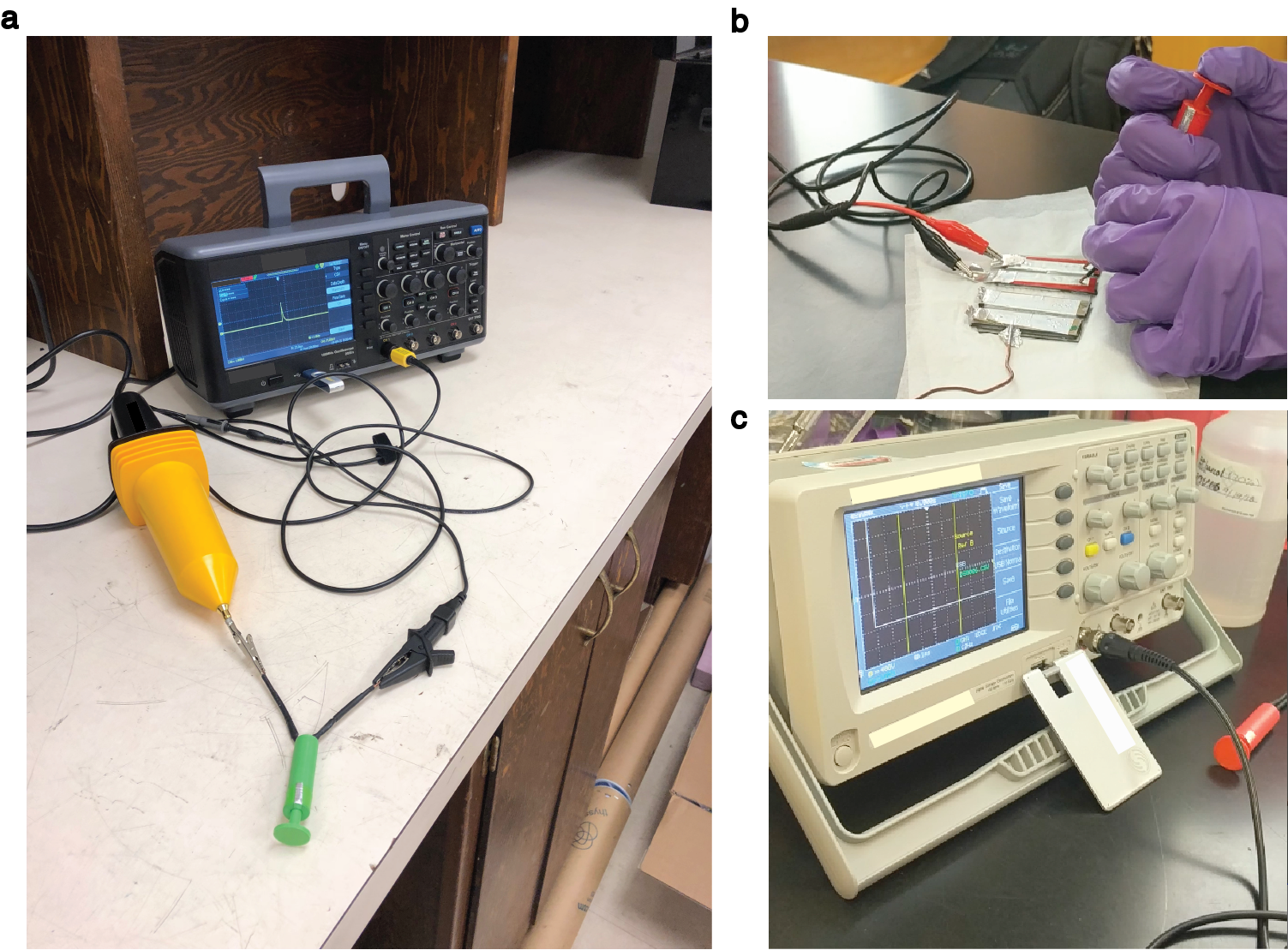

Supplement: S11 Fig — (a) The oscilloscope was connected to a high-voltage probe along with the ElectroPen. (b) Oscilloscope connections to the cuvette while running the trials to ensure voltage passed through cell suspension. (c) Sample waveform obtained from oscilloscope during trial (voltage clipping is present as ElectroPen voltage output exceeds capacity of the oscilloscope). (TIF) [file pbio.3000589.s011.tif]

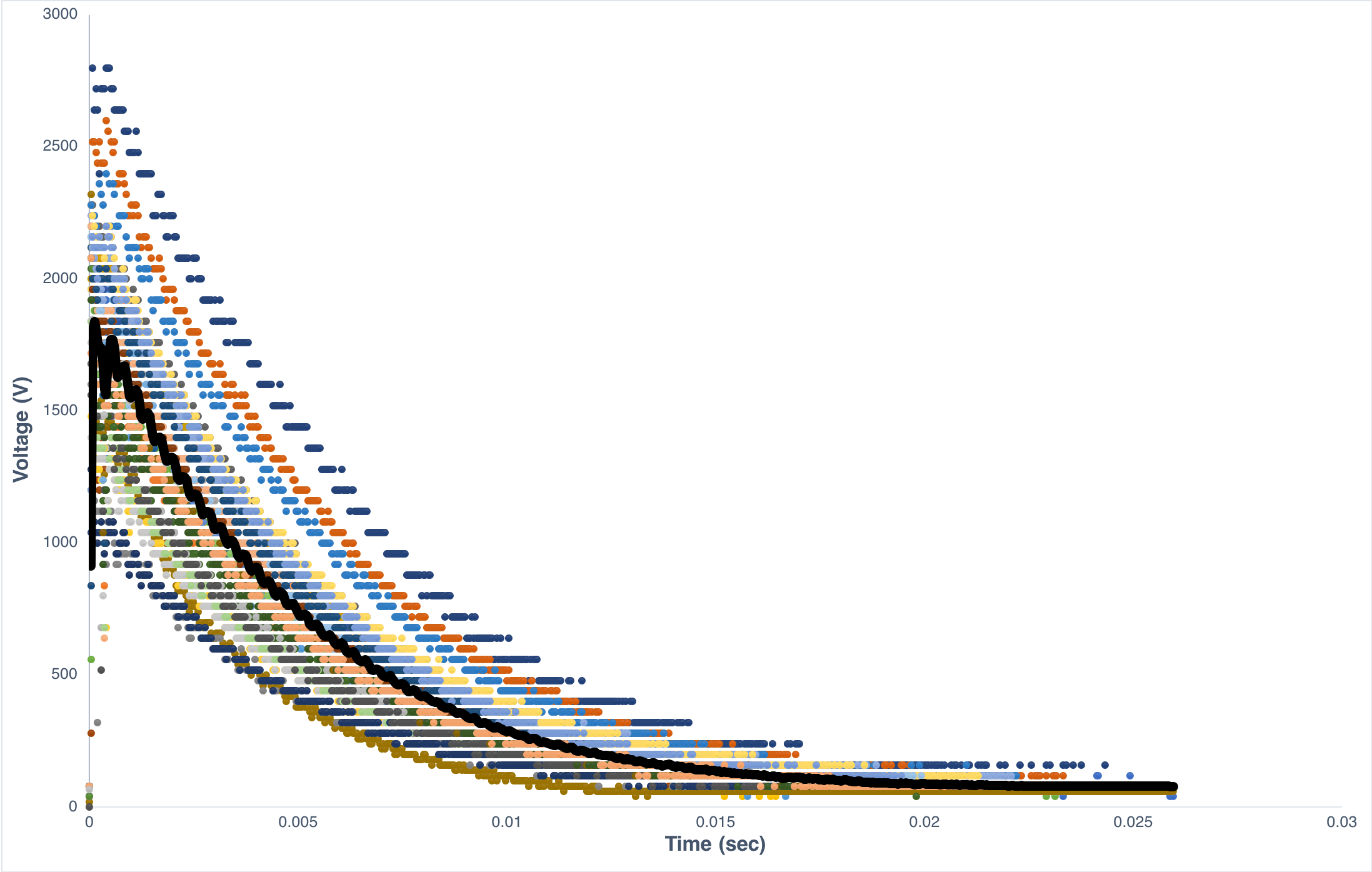

Supplement: S12 Fig — The solid black line represents the average value for the decay pulse (without curve fitting), and the scatter points represent individual values. The data for S12 Fig can be found on GitHub under the S1 Data file, under the sheet titled Fig 2A. (TIF) [file pbio.3000589.s012.tif]

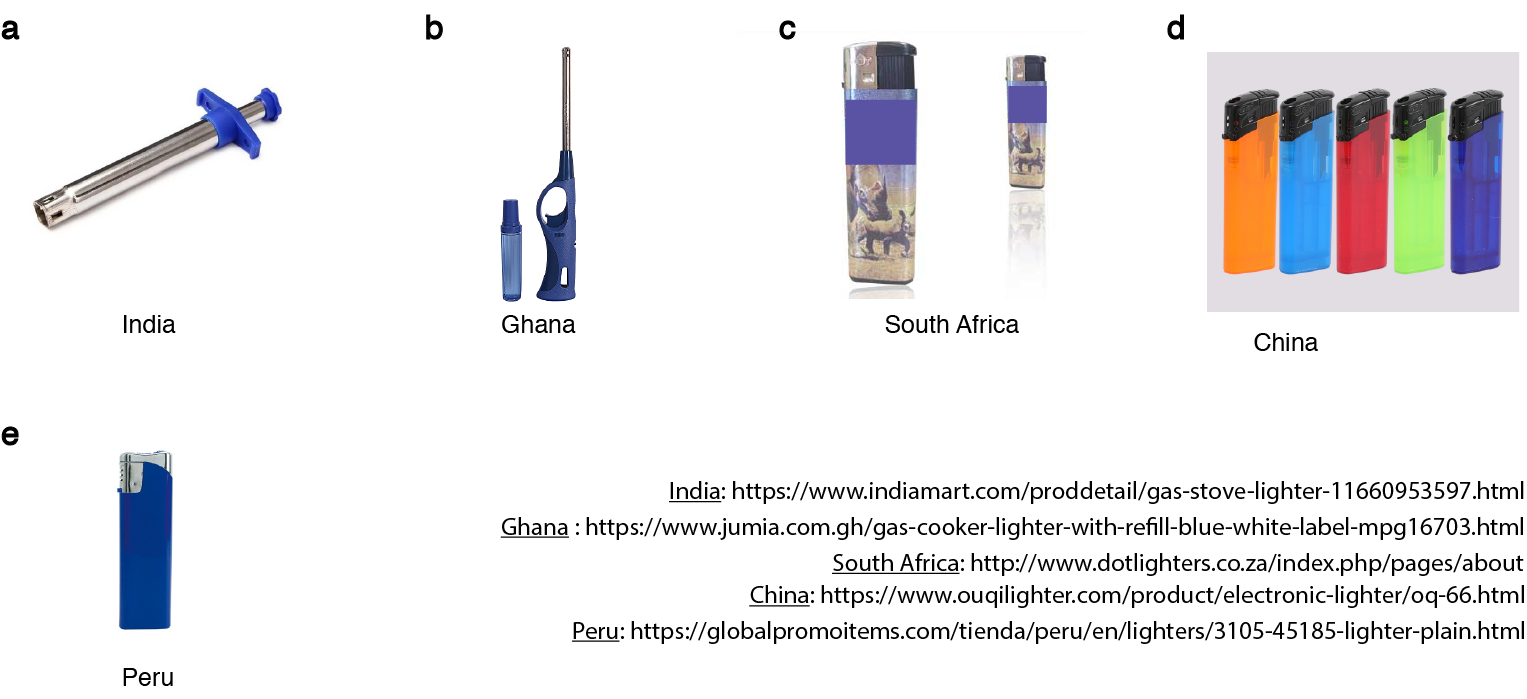

Supplement: S13 Fig — Indicative of how the overall mechanism and construction of lighters remains consistent across the world. (TIF) [file pbio.3000589.s013.tif]

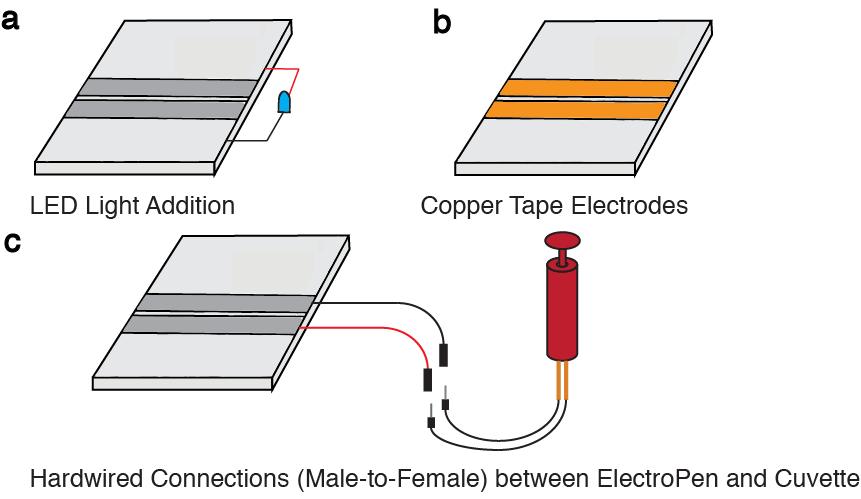

Supplement: S14 Fig — (a) Potential setup for LED light indicator to provide identification for successful electroporation. (b) Potential copper electrodes for millifluidic channels instead of aluminum tape electrodes. (c) Potential hardwired version of ElectroPen. LED, light-emitting diode. (TIF) [file pbio.3000589.s014.tif]
