# Supplementary material for: ElectroPen: An ultra-low–cost, electricity-free, portable electroporator
Source: PLoS Biol. 2020 Jan 10;18(1):e3000589. doi: 10.1371/journal.pbio.3000589 (PMC6953602; doi:10.1371/journal.pbio.3000589)
Supplement: S1 Text — (PDF) [file pbio.3000589.s019.pdf]

Supplementary Information
ElectroPen: An ultralow-cost, electricity-free, portable electroporator

I. MATERIALS AND METHODS

A. ElectroPen System

The mechanism utilized for the production of the voltage output has been obtained from a conventional stove lighter gun. The hammer action mechanism present for the ignition of butane gas has been extracted from the lighter and placed into a 3D-printed casing to allow for greater ease of operation and stability. The CAD (.stl) file for the casing can be found on Github, https://github.com/bhamlalab/electropen. A toggle was added to assist in the exertion of a force on the hammer action that strikes the crystal, and copper wires were attached for conductance and extension of the length of the terminals. Additionally, a 0.1 cm millifluidic channel was designed to serve as a less expensive alternative for standard electroporation cuvette. It consists of two pieces of acrylic with aluminum (or copper) tape on the ends to create a gap distance for the electrical arc. This is surrounded by another piece of acrylic to hold the structure in place and create a channel for the cell solution to be held. Other materials can be easily substituted in place of acrylic or glass for fabrication.

B. Recombinant Plasmid

The plasmid (pADS001) used is a medium copy plasmid (p15A origin) with chloramphenicol resistance. pADS001 constitutively expresses super folder green fluorescent protein (sfGFP) under a strong promoter (J23119 from the Parts Registry) and a strong ribosomal binding site. The plasmid is purified with Omega EZNA miniprep kit according to the manufacturer's protocol, and proper sequence of the expression cassette is confirmed via DNA sequencing.

C. Electrocompetent Cell Preparation

BL21 *E. coli* cells were inoculated into 5 mL overnight liquid cultures and stored in an 37°C incubator set to shake at 170 rpm. After 18 hours of growth, they were diluted to a ratio of 1:100 in sterile Luria Bertani (LB) medium and shaken in a 37°C incubator at 170 rpm. Autoclaved water and 10% glycerol solution were then stored in a refrigerator at 4°C in preparation for the following wash steps. The Optical Density (OD) of the cells was continuously monitored every 30 minutes until the cells reached an OD of approximately 0.6. The cells were then transferred into 50 mL conical tubes on ice and allowed to cool for 10 minutes. The suspension was centrifuged at 2500 x g and 4°C for 6 minutes, after which the supernatant was discarded. The cell pellets were combined by serially resuspending in 13 mL of chilled autoclaved water to concentrate the cell suspensions into two 50 mL conical flasks. The suspension was centrifuged at 2500 x g and 4°C for 6 minutes, after which the supernatant was discarded. The previous wash step was then repeated. The cell pellets were then resuspended in 13 mL of chilled 10% glycerol solution. The suspension was centrifuged at 2500 x g and 4°C for 6 minutes, after which the supernatant was discarded. The previous wash step was then repeated. The cell pellets were then resuspended and combined in 4 mL of chilled 10% glycerol and aliquoted into chilled microcentrifuge tubes with 100 μL per tube. They were then stored in a -80°C freezer for long-term preservation. Additional options for storage in areas without access to such equipment can include storage in liquid nitrogen or temporarily on dry ice for periods of up to 24-48 hours. Alternatively, the cells can be prepared in a manner such that they are used right after being prepared.

D. Electroporation

Millifluidic channels were stored in a -20°C freezer for at least 24 hours before electroporation. The custom millifluidic channel was then taken out of the freezer and sterilized with ethanol before electroporation. During this time, the millifluidic channel was kept on ice. The ElectroPen was then tested on the empty millifluidic channel to ensure that the spark successfully traveled between the electrodes. The frozen DH5a or BL21 electrocompetent cells thawed on ice for approximately 10 minutes, after which they were aliquoted in 50 μL amounts to individual ice-chilled tubes. 1 μL (2 ng DNA) of the plasmid construct was then added to each cell suspension and tubes were gently resuspended using a pipette to ensure thorough mixing while water was added to a different cell suspension as the negative control. The cell suspension was then transferred into the electrode gap and shocked 1-10 times with the ElectroPen. Immediately following the shock, 100 μL of pre-warmed (37°C) Luria Bertani broth was carefully added to the gap to recover the cell suspension. The recovered suspension was then transferred into a microcentrifuge tube, after which an additional 900 μL of Luria Bertani broth was added to the tube for recovery. The tubes were stored at 37°C in an incubator set to shake and allowed to recover for 60-90 minutes. The tubes were centrifuged at 3000 x g for 1 minute, and 800 μL of the supernatant was discarded. The cell pellet was then reconstituted in the remaining LB media and plated onto the appropriate

antibiotic plates.

For electroporation with the Biorad Micropulser, unopened cuvettes were removed from their packaging and placed into a bucket of ice. The cell suspensions were then prepared in the same manner as mentioned above. The electroporator was set to 1.8kV and 5.0ms while the cuvettes channels chilled and cells thawed. The above mentioned amount was then transferred into the cuvette channel and placed into the electroporator. The pulse was then applied by pushing the appropriate button, and 1 mL of warmed Luria Bertani broth was added to the cuvette and cells were resuspended. They were then transferred into new tubes and shaken according to the same protocol mentioned above.

E. Efficiency Calculations

The plates were allowed to incubate for 24 hrs and then taken out to manually count colonies. A picture of the plate was analyzed using Adobe Illustrator, where colonies were tracked and followed with an object count. Transformation efficiency was then calculated as number of colonies divided by ng of DNA plated * 1000 to convert to colony forming units per microgram of DNA.

F. Fluorescence Measurements

For *in vivo* fluorescence measurements, three colonies were picked from each of the test cases: a no plasmid control with just water, plasmid introduced by electroporator, and plasmid introduced by electropen. The colonies from no plasmid controls were inoculated into 5 mL of LB media without antibiotic resistance, and the colonies bearing pADS001 plasmid were inoculated into 5 mL of LB containing chloramphenicol. The cultures were grown at 37°C and 170 rpm for 18 hrs before 50 μL of the overnight culture were transferred into 950 μL of M9 minimal media (1xM9 minimal salts, 0.01% thiamine hydrochloride, 20 mM Dextrose, 2 mg/mL SC-Ura, 2 mM MgSO4, 0.1 mM CaCl2) containing chloramphenicol antibiotics. Overnight cultures of no plasmid controls were transferred into M9 without antibiotics. The M9 subcultures were then grown for 4 hours at 37° and 180 rpm. After 4 hours, 100 μL of each subculture was transferred onto a clear bottom 96 well plate (Corning 3651) and diluted with 100 μL of 1xPBS. Optical density (OD_{600} nm) and fluorescence (485 nm excitation and 528 nm emission, Gain 60) were measured on a Biotek Synergy H4 microplate reader at room temperature. All experiments were repeated independently three times for a minimum of nine biological replicates per test case.

G. Voltage Measurements

A high voltage probe (Elditest CT4026) with a 1000:1 divider ratio was used with an oscilloscope (Teledyne Lecroy WaveAce2014) with a measurement span of 25 ms, and with a vertical scale of 1 kV to measure the output voltage from the ElectroPen (Supplementary Figure S11). The high voltage probe has a grounding wire which was connected to the oscilloscope.

H. Replicate Samples

Fluorescence measurements were made on biological replicate samples. Colonies from each plate for each trial were isolated and grown in individual liquid cultures, and this was done for three separate trials. The trial average is a combination of the replicates for each trial and average for the sample is the average of the trials for that sample (negative control, electroporator, and ElectroPen).

I. Organization of Experimental Trials

For the data represented in Fig. 4 (a,b), the trials were conducted in subsets. The trials were conducted in two batches run across different days. The ElectroPen samples were run first, followed by the electroporator, and then finally the negative control.

For the data represented in Supplementary Fig. S8, the 3 ElectroPen experimental samples were run first followed by the positive control with the electroporator and negative control with the ElectroPen. This trial was conducted at the University of Georgia with the support of UGA iGEM team members in an effort to characterize the ElectroPen's functionality with the DH5a strain of *E. coli*. The experiments were conducted on DH5a E. coli using the BioRad Micropulser with the same protocol listed in the methods section. The data shown above represents n=3 for both the electroporator and ElectroPen, with error bars indicative of standard deviation of the replicates of the samples.

J. TAS Taipei Plasmid Construct

The plasmid utilized encoded GFP (BBa E0040) under the control of a pLac promoter (BBa R0010) with parts obtained from the iGEM Parts Registry.
