# Supplementary material for: ElectroPen: An ultra-low–cost, electricity-free, portable electroporator
Source: PLoS Biol. 2020 Jan 10;18(1):e3000589. doi: 10.1371/journal.pbio.3000589 (PMC6953602; doi:10.1371/journal.pbio.3000589)
Supplement: S2 Text — (PDF) [file pbio.3000589.s020.pdf]

Supplementary Information
ElectroPen: An ultralow-cost, electricity-free, portable electroporator

I. SUPPLEMENTARY THEORY DISCUSSION

A. Estimation of theoretical voltage for piezoelectric crystal

For a piezoelectric crystal, the theoretical V_{th} can be predicted from the following equation:

$$V_{th} = \frac{(d_{33})(F)(t)}{(\epsilon_r)(\epsilon_0)(A)}, \quad (1)$$

where d_{33} = piezoelectric charge coefficient, F = force applied on the crystal, t = thickness of the crystal, ϵ_r = relative permittivity, ϵ_0 = permittivity of a vacuum, and A = area of impact surface [1]. For a given ElectroPen design, all the parameters, including force applied on the crystal, remain constant. Using published values for the parameters for a PZT crystal and a force (F = 10 N) , we obtain $V_{th} \sim 2.7$ kV , which is of the same order of magnitude as the experimentally measured voltages.

The piezoelectric crystal present within the lighter consists of a disc-orientation, with voltage enhanced through longitudinal thickness. The force is applied on the bottom surface, with the thickness (in this case height) of the crystal acting as the amplification factor (Supplementary Fig. S10). The following equations were utilized to calculate the theoretical maximum voltage of the piezoelectric crystal present for the ElectroPen [2].

$$D_3 = (d_{33})(\sigma_1),$$

where D_3 refers to the polarization of the crystal, d_{33} represents the piezoelectric charge constant, and σ_1 is the stress on the plate. Substituting variables to include charge Q, force F_1, and area A_1, we obtain

$$\frac{Q}{A_3} = (d_{33})(\frac{F_1}{A_1}).$$

We can then substitute charge Q for capacitance C_3 and voltage V_3, and rearrange to obtain

$$(C_3)(V_3) = (d_{33})(F_1)(\frac{A_3}{A_1}).$$

We incorporate the definition of capacitance, $C = \frac{\epsilon_0 \epsilon_r A_3}{t}$ and re-arrange to obtain,

$$V_3 = \frac{(d_{33})(F_1)(t)}{(\epsilon_0)(\epsilon_r)(A_1)}.$$

We now substitute the piezoelectric voltage constant definition $g_{33} = \frac{d_{33}}{(\epsilon_0)(\epsilon_r)}$, to obtain the final expressions

$$V_{th} = V_3 = \frac{(g_{33})(F_1)(t)}{A_1}.$$

Using values of g_{33} = 0.0265Vm/N [3], F_1 = 10 N (measured force, see main text), t = 8 mm, and A = $\pi * r^2$ where r = 1 mm, we obtain a maximum theoretical output of 2,699.3 V, which is of the same order of magnitude as experimental voltages. The small mismatch in the experimental and theoretical values is attributed to the resistance in the copper wires as well as confinement effects of the crystal within the plastic case.

[1] Porcelli EB, Filho VS. Induction of Forces at Distance Performed by Piezoelectric Materials. Journal of Power and Energy Engineering. 2018;6:33–50. doi:10.4236/jpee.2018.61004.

[2] Fundamentals of Piezoelectricity. In: Piezoelectric Transducers for Vibration Control and Damping. London: Springer; 2006. p. 9–35. Available from: https://pdfs.semanticscholar.org/f063/529f370fa281407ba82e5803098940c0ba26.pdf.

[3] APC International L. Piezoelectric Ceramics: Principles and Applications. APC, International, Ltd.; 2011. Available from: https://www.americanpiezo.com/knowledge-center/piezo-theory/piezoelectric-constants.html.
