# Supplementary material for: ElectroPen: An ultra-low–cost, electricity-free, portable electroporator
Source: PLoS Biol. 2020 Jan 10;18(1):e3000589. doi: 10.1371/journal.pbio.3000589 (PMC6953602; doi:10.1371/journal.pbio.3000589)
Supplement: S1 Table — The listed electroporators reflect common equipment utilized in labs. The ElectroPen reflects a fraction of the cost of its industrial equivalent while not requiring access to electricity and weighing magnitudes less. *0.1/0.2-cm gap industrial electroporation cuvettes. †The cost includes only the device. (PDF) [file pbio.3000589.s021.pdf]

Supplementary Information
ElectroPen: An ultralow-cost, electricity-free, portable electroporator

Device	Additional Supplies	Cost†	Electricity	Weight
BioRad Micropulser Electroporator	Commercial cuvettes*	$2,399.00	Yes	2.9kg
Eppendorf Eporator	Commercial cuvettes*	$2,619.00	Yes	3.2kg
BioRad Gene Pulse Cell Microbial Electroporator	Commercial cuvettes*	$6,875.00	Yes	6.6kg
**ElectroPen**	**Low-cost, custom cuvettes**	**$0.23**	**No**	**13g**

TABLE I. Comparison between standard commercial electroporators and the ElectroPen. The listed electroporators reflect common equipment utilized in labs. The ElectroPen reflects a fraction of the cost of its industrial equivalent, while not requiring access to electricity and weighing magnitudes less.*0.1/0.2 cm Gap Industrial Electroporation Cuvettes. † cost includes only the device.
