# Supplementary material for: ElectroPen: An ultra-low–cost, electricity-free, portable electroporator
Source: PLoS Biol. 2020 Jan 10;18(1):e3000589. doi: 10.1371/journal.pbio.3000589 (PMC6953602; doi:10.1371/journal.pbio.3000589)
Supplement: S2 Table — The protocols for electroporation for these organisms can be found at the respective links attached. (PDF) [file pbio.3000589.s022.pdf]

# Supplementary Information
# ElectroPen: An ultralow-cost, electricity-free, portable electroporator

| Organism/Cell Line | Voltage (kV) | Time Constant (ms) | Source |
|---|---|---|---|
| *Escherichia coli* | 1.8 | 5.0 | *E. coli* electroporation protocol [1] |
| *Saccharomyces cerevisiae* | 2.0-2.25 | 6.0 | *S. cerevisiae* electroporation protocol [2] |
| HeLa | 0.220 | 25 | HeLa electroporation protocol[3] |
| *Staphylococcus aureus* | 1.8 | 2.5 | *S. aureus* electroporation protocol [4] |
| *Saccharomyces pombe* | 2.0 | 5.0 | *S. pombe* electroporation protocol [4] |

TABLE I. List of different time constants and peak voltages optimized for the electroporation of different organisms. The protocols for electroporation for these organisms can be found at the respective links attached.


[1] Gonzales MF, Brooks T, Pukatzki SU, Provenzano D. Rapid protocol for preparation of electrocompetent Escherichia coli and Vibrio cholerae. Journal of Visualized Experiments. 2013;(80). doi:10.3791/50684.

[2] Delorme E. Transformation of Saccharomyces cerevisiae by Electroporation; 1989. 9.

[3] Protean. Elpo-HeLa;. Available from: https://www.protean.bio/doc/transfection/Elpo-HeLa.pdf.

[4] Bio-Rad. MicroPulser ™ Electroporation Apparatus Operating Instructions and Applications Guide Catalog Number 165-2100;. Available from: https://www.bio-rad.com/webroot/web/pdf/lsr/literature/4006174B.pdf.
